# Editorial: The GABAA receptor: a target of pharmacologically active molecules

**DOI:** 10.3389/fphar.2024.1440937

**Published:** 2024-06-18

**Authors:** Margot Ernst, Wei Lu, César Mattei

**Affiliations:** ^1^ Department of Pathobiology of the Nervous System, Center for Brain Research, Medical University of Vienna, Vienna, Austria; ^2^ National Institute of Neurological Disorders and Stroke, National Institutes of Health, Bethesda, MD, United States; ^3^ University Angers, CarMe, Unité MITOVASC, UMR CNRS 6015, INSERM U1083, SFR ICAT, Angers, France

**Keywords:** GABA_A_ receptors, subunit, therapeutics, epilepsy, central nervous system

The homeostasis of the central nervous system is ensured by a balance between excitation and inhibition. While the excitatory neurotransmitter systems are mainly driven by glutamate, central inhibition is largely dominated by GABAergic transmission. GABA_A_ receptors (GABA_A_Rs) are pentameric ionotropic receptors whose central pore acts as an ion channel that dissipates Cl^−^ and HCO_3_^−^ currents. Thus, they provide rapid neuronal inhibitory neurotransmission in most areas of the brain and spinal cord. They have long been therapeutic targets for drugs that facilitate central inhibition, including anxiolytics, muscle relaxants, anticonvulsants, hypnotics, anaesthetics and sedatives. While the stoichiometry of these receptors is well described - the classic organization is 2α+2β+γ/δ - the high-resolution structure of some of them was elucidated just a few years ago, paving the way for a better understanding of GABA_A_R pharmacology, and for the future development of therapeutic agents specific to the many subtypes of these receptors. By now, various different subunit arrangements have been identified (Sente et al., 2022).

Given the close relationship between structure and function inherent in ligand-gated ion channels (LGICs), GABA_A_Rs can be positively or negatively modulated by different classes of endogenous molecules - such as steroids - or exogenous molecules - such as insecticides or neurotoxins. Their subunit composition governs the affinity of a ligand for these receptors. For example, it is well established that benzodiazepines bind preferentially to receptors with a γ subunit.

In this Research Topic, several articles have looked at the pharmacology of GABA_A_Rs, particularly from a therapeutic perspective. Firstly, it is well established that GABA_A_Rsare at the heart of certain forms of epilepsy (Richardson et al.). In fact, different drugs that positively modulate GABA_A_R activity are both short- and long-term treatments for human epilepsy. The study by Nakakubo et al. examined the effects of KRM-II-81, a positive allosteric modulator (PAM) of α2/α3-GABA_A_Rs, on an animal model of Dravet syndrome, a severe form of childhood epilepsy. They used mice carrying a loss-of-function mutation in the Scn1A gene encoding the Nav1.1 sodium channel expressed in the central nervous system. In a hyperthermia-induced seizure challenge designed to assess the threshold for triggering seizures, KRM-II-81 induced a significant increase in this threshold. In addition, this compound exerts anxiolytic and sedative effects, most probably linked to its allosteric activity on GABA_A_Rs. These effects may be seen as beneficial in the management of epileptic seizures. Finally, from a mechanistic point of view, KRM-II-81 potentiates inhibitory post-synaptic currents in hippocampal CA1 pyramidal neurons, an effect which is enhanced in mice carrying the deleterious Nav1.1 mutation. This work highlights the value of KRM-II-81 in the management of patients with Dravet syndrome.

Fenamates are broad-spectrum non-steroidal cyclooxygenase (COX) inhibitors: they are used for their anti-inflammatory, analgesic and antipyretic activities. However, they also target β2/β3-GABA_A_Rs as PAMs (Halliwell et al., 1999). In the study published by Salmanzadeh and Halliwell, the Fenamates proved effective in reducing the excitability of human neuroglial-differentiated stem cells. More specifically, the four Fenamates studied reduced the frequency of action potentials induced by the addition of 4-aminopyridine in a dose-dependent manner. This effect was not due to inhibition of COXs, as it was not found with non-fenamate NSAIDs such as ibuprofen. This *in vitro* study therefore suggests the use of Fenamates as potential anti-epileptics.

In view of the involvement of GABA_A_Rs as a validated therapeutic target in epilepsy, the recent elucidation of their structure provides fresh impetus for the design of specific molecules that would limit undesirable effects. This is the point of view developed in the review by Richardson et al.. The pharmacology of GABA_A_Rs is presented, with the recent identification of molecular partners of the receptors (LHFPL4, Cltpm1 and Shisa7), their synaptic or extra-synaptic expression and the genetic variants responsible for epilepsy. From a therapeutic point of view, the authors recall the drugs that have been well characterized for their anti-epileptic properties. The development of effective drugs will probably follow several guidelines, including the specificity of a PAM for a particular GABA_A_R subtype (incorporating the α2, α3 or α5 subunits). Gene therapy is also envisaged to restore normal expression of defective channels (notably Nav1.1 in Dravet syndrome) and increase GABA_A_R transmission.

While restoration of GABergic transmission is a major process in the management of epilepsy, enhanced GABAergic neurotransmission can lead to severe pathologies such as hepatic encephalopathy (HE) with hyperammonemia. In these diseases, NH_4_^+^ ions are metabolised by CNS astrocytes for the synthesis of glutamine, thanks to glutamine synthetase. Excess glutamine can lead to the development of cerebral oedema and death. Alterations in GABAergic neurotransmission are an important factor in the cognitive and motor dysfunctions observed in HE and hyperammonemia. Llansola et al. review the role of GABA networks in the cerebellum in these two pathologies, which may be linked. The release of inflammatory mediators, primarily TNFα, is thought to indirectly induce this GABAergic activation. Thus, neuroinflammatory and GABA transmission mechanisms appear to be highly intertwined. Inhibition of GABA_A_R activity using antagonists or allosteric modulators is a promising avenue for reducing inflammation and its consequences in HE and hyperammonemia.

Menzikov et al. propose a pharmacological view of phenols in relation to GABA_A_Rs in their review, highlighting their toxicity or potential therapeutic benefits. The versatility of the effects of phenols on GABA_A_Rs - from activation to inhibition - is probably explained by the molecular heterogeneity of what are known as phenols, i.e., compounds consisting of an aromatic hydrocarbon ring and one or more hydroxyl groups. Given their ability to cross the blood-brain barrier, they are likely to induce effects in people who are exposed to them. The review also emphasizes the close relationship between the structure and function of these receptors: the subunit composition determines the pharmacological activity of the ligands.

This ligand/receptor pharmacological approach is also being used to investigate the effects of taurine and homotaurine (HT) on cerebellar neurons. Meera et al. highlight the structural analogy between GABA and both amino acids in their study, pointing out that HT - as conjugated to valine - is currently a drug candidate in the treatment of Alzheimer’s disease. Acetyl-HT is also a drug used in the treatment of alcoholism, a disease in which GABA_A_Rs play a predominant role. The study by Meera et al. shows a concentration-dependent effect of taurine and HT on cerebellar granule cells, using an electrophysiological approach. Both amino acids induce an agonist effect on GABA tonic current. These results were confirmed by binding tests on mouse brain native GABA_A_Rs, in which taurine and HT displaced the binding of their agonist muscimol.

Finally, Müller et al. also used muscimol to investigate the ability of various antagonists to displace its binding to GABA_A_R orthosteric site as a function of brain region. GABA_A_Rs are present throughout the brain, but certain combinations dominate depending on the area. The study therefore used muscimol as a probe to characterize the level of interaction of antipsychotic agents (clozapine, loxapine and chlorpromazine) with cerebral GABA_A_Rs. The results indicate that, depending on the ligand and the area studied, the muscimol binding displacement is competitive or partly allosteric. The muscimol binding site varies widely in each brain area, confirming that there are many differentially expressed GABA_A_R populations. These receptor clusters bind differently to the antipsychotic agents used. This illustrates the ability of therapeutic agents to exert significant effects on the central nervous system.

The diversity of subjects covered in this topical Research Topic testifies to the crucial role of GABA_A_Rs as both therapeutic targets and regulators of the brain’s physiological equilibrium. Numerous studies have used the subunit composition of these receptors as a starting point for the pharmacological characterization of ligands. Given the specific expression of certain subunits in precise areas of the brain and our knowledge of the structure of human receptors, the development of highly selective drugs is now a medium-term prospect. This topical Research Topic has sought to illustrate this structure-function relationship, and the published papers will add to our knowledge in this area.
